# A Case of Ichthyosis

**Published:** 1839-07-13

**Authors:** B. D. Neill

**Affiliations:** Philadelphia


### A CASE OF ICHTHYOSIS,

By B. D. Neill, M. D.

At the close of the month of May, 1839, I was called to visit C. P., a white female, æt. 80 years, a washerwoman, resident in the Western District of Southwark, Philadelphia, who was suffering from ichthyosis.

The patient had been attended by me during the winter, for an ulcer on the spine of the tibia of the left side, apparently involving the periosteum and superficial lamina of the bone. The ulcer, when the history of the case commences, was covered with a cicatrix.

The general health during life has been good, and there had been but slight constitutional disturbance during the progress of the ulcer.

On the afternoon of May 27th, the leg of the same side, from the toes to the knee joint, appeared œdematous, shining and tense, of scarlet colour, with numerous patches of elevated brownish scales scattered over the surface, and there was present stinging, burning pain in the part affected. After the lapse of a few days the surface exhibited extensive cracks, from which an ichorous discharge flowed; there was tumefaction of the ancle. The treatment has been as follows, and in the order indicated: Leeches, freely applied to the more inflamed portions of the leg and foot; rye meal, sprinkled dry upon the moist patches of the surface; tepid water, solutio plumbi subacet.; flaxseed and corn meal poultices. Simple cerate, spread on linen, was next used, and followed by a bandage extending from the toes to the knee, which was kept wet with cold water. At night, pul. ipecac. comp. [chart. ij $\overline{aa}$ gr. v.] Attention to elevated position of the limb was enforced; occasional purgation and low regimen were also directed.

After one month, desquamation is completed, the swelling has subsided, and a fresh, blushing, translucent epidermis is formed.

N. B.—The *right* leg exhibits now the distinctive *scaly* appearance, but with no fissures, swelling or discharge. The arms also present a similar appearance.

For a description of this disease see Plumbe on the Skin, in Bell's Select Medical Library.

*Philadelphia*, July 3d, 1839.

